# Embryonic origin of cancer in newborn twins

**DOI:** 10.1038/s41467-026-78178-5

**Published:** 2026-10-06

**Authors:** Barbara Walkowiak, Sadie Baskind, Manas Dave, Pooja Balasubramanian, Nathaniel Anderson, Jonathan Kennedy, Thomas R. W. Oliver, Rohan Verma, Sarita Depani, Simon Hannam, Tom Watson, Dyanne Rampling, Karin Straathof, Liina Palm, J. Ciaran Hutchinson, Henry Lee-Six, Olga Slater, Sam Behjati

**Affiliations:** 1 Wellcome Sanger Institute, Hinxton, UK; 2 Great Ormond Street Hospital NHS Foundation Trust, London, UK; 3 Great Ormond Street Biomedical Research Centre, London, UK; 4 Department of Pathology, University of Cambridge, Cambridge, UK; 5 Cambridge University Hospitals NHS Foundation Trust, Cambridge, UK; 6 UCL Cancer Institute, London, UK; 7 Present Address: Department of Paediatrics, University of Cambridge, Cambridge, UK

**Keywords:** Cancer genomics, Developmental biology, Embryonal neoplasms

## Abstract

Studying monozygotic twins who present with identical cancers informs on the developmental origins of childhood tumours. Here, we perform whole genome sequencing on multiple tumour, normal, and placental samples to reconstruct the phylogeny of a soft tissue cancer that spread *in utero* between monozygotic twins. We demonstrate the origin of the tumour from one twin and time its diversification and transplacental spread to the other twin, showing that the spread occurred only once. This generalisable and scalable approach additionally allows us to dissect the earliest stages of twinning, revealing unexpected asymmetrical contributions of embryonic lineages to the placenta and each twin and challenging the classical model of twinning.

## Abstract (web summary)

Identical twins with the same cancer can reveal how childhood tumours arise and spread before birth. Here, the authors use whole-genome sequencing of tumour, normal and placental tissues to show that a sarcoma that originated in one twin spread to the other *in utero*, and trace early embryonic lineages that contributed differently to the twins and placenta.

## Introduction

There are exceptional reports of identical twins (i.e. derived from the same zygote) who present with the same cancer^1,2^. Tumours in twins may arise independently, in particular in the context of a cancer-predisposing mutation in the germline^3^. Alternatively, they may result from *in utero* transfer of tumour cells from one twin to the other^4,5^. These scenarios may be distinguished by comparing the genetic changes in the two tumours: shared variants indicate a single origin followed by spread, while independent sets of mutations imply separate origins. Healthy cells also acquire somatic mutations over time, and so by looking at the pattern of sharing between the tumour and different normal samples, it is possible to reconstruct not only the life history of the tumour but also some of the earliest steps in development^6–9^.

**Fig. 1:**
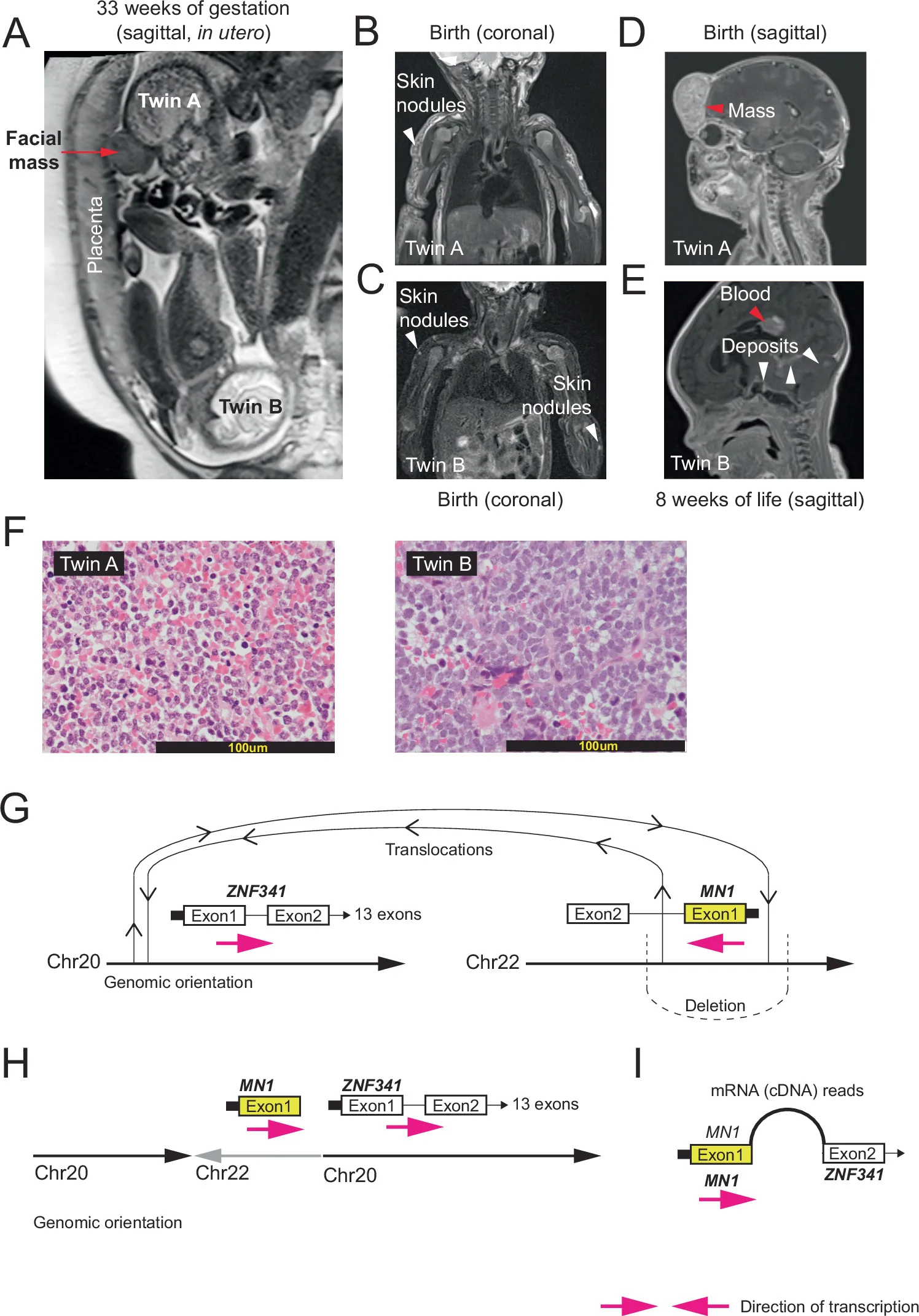
Presentation of twins with a sarcoma. **A** Shows a fetal MRI at 33 weeks gestation, 1 week before the twins were delivered. A facial mass was observed in twin A. **B**, **C** Show coronal MRI images of twins A and B, respectively at birth. Both had skin nodules. **D** Shows the facial mass of twin A at birth. **E** Shows the intracranial haemorrhage (red arrow) noted in Twin B at 8 weeks of age, along with leptomeningeal tumour deposits (white arrows). **F** Represents haematoxylin and eosin-stained sections of skin deposits in twin A and B, respectively, with identical small round blue cell morphology noted. One representative histological section chosen by a consultant pathologist after scanning the whole slide is shown for each tumour. **G** Presents the translocation that led to the formation of the *MN1::ZNF341* fusion. **H** Depicts the gene fusion that was found to be driving the tumours. **I** Shows the resulting cDNA derived from RNA sequencing.

Here, we use this approach to investigate the origin of lethal cancers in female monozygotic twins^10^. During fetal imaging, a facial mass had been found in one girl (hereafter referred to as twin A), leading to an earlier delivery. At birth, tumour lesions were found to be disseminated both in twin A and her sister (twin B) (Fig. 1A–E and Supplementary Note 1). While twin A had tumour deposits in multiple organs, twin B’s lesions were found at presentation in the skin, with leptomeningeal disease detected later. Biopsies of their tumours were reported as *MNF1*::*ZNF341* rearranged undifferentiated sarcomas^11^, with all tumour samples from both children bearing this driver fusion (Fig. 1F and Supplementary Note 1). Both twins, sadly, died soon after birth, and their family consented to post-mortem examinations for research. In this work, we first reconstruct the early lineages contributing to both twins and the placenta. We next use this reconstruction to trace the origin and spread of the tumour.

## Results

### Phylogeny of normal twinning

To reconstruct the phylogeny of normal twinning and of cancer formation and spread, we collected normal and tumour tissues of both twins obtained at post-mortem. We performed whole-genome sequencing on 23 normal samples (six normal samples from each twin, and 11 placental samples) and 10 tumour samples (eight from twin A, two from twin B) (Supplementary Data 1). In addition, we sequenced 12 placenta samples enriched for trophoblast by laser capture microdissection (LCM). We called all classes of mutations and identified developmental somatic substitutions, i.e. mosaic variants that are shared between some cells across both twins and the placenta. We additionally performed RNA sequencing to resolve and validate the *MN1::ZNF341* fusion (Fig. 1G–I, Supplementary Fig. 1 and Supplementary Data 2).

**Fig. 2:**
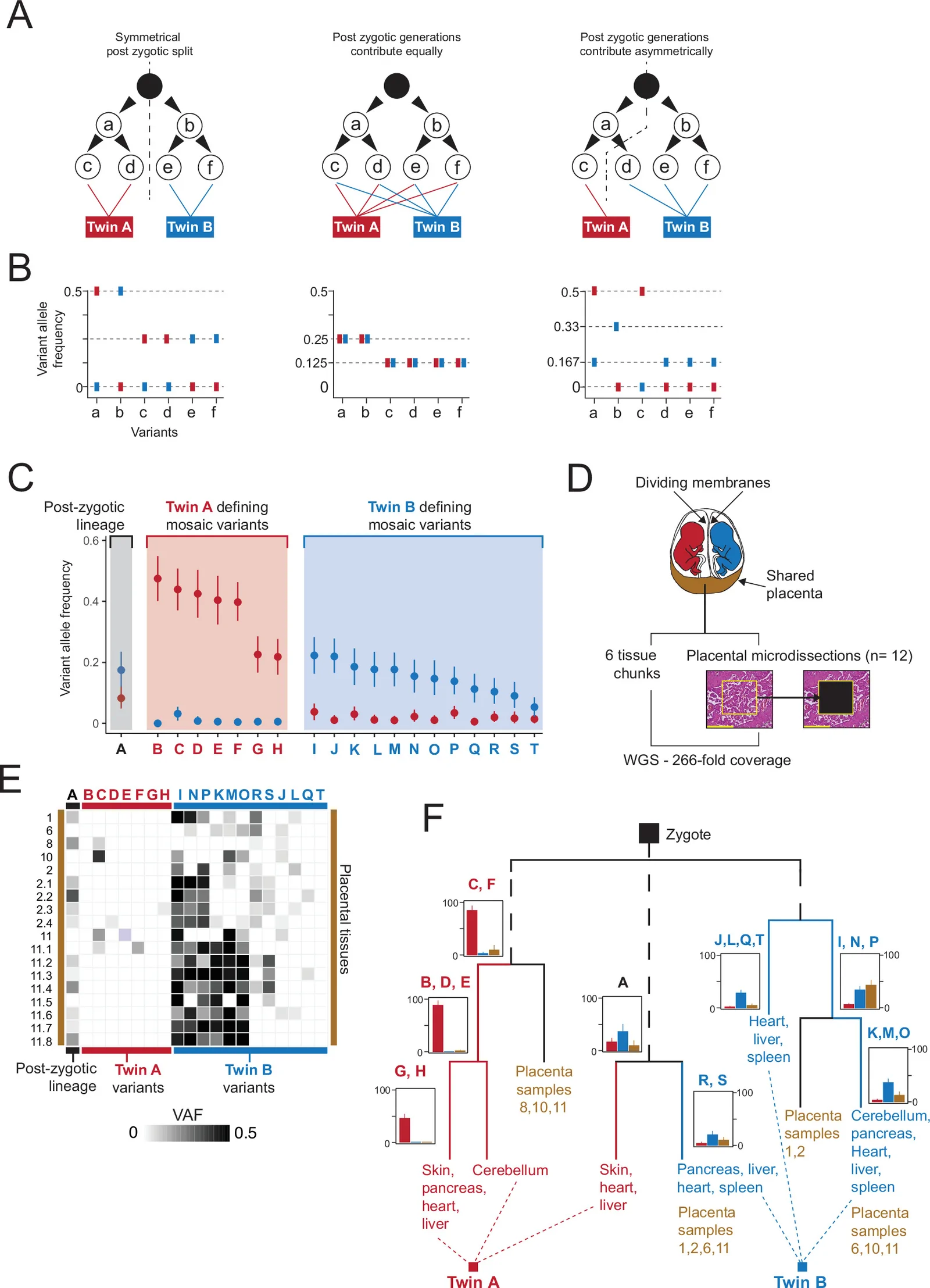
Reconstruction of early development. **A** Presents three different possible scenarios of the early events associated with twinning. **B** Presents the expected variant allele frequencies (VAFs) of early embryonic mutations under each scenario. **C** Presents VAFs of early embryonic mutations in normal samples of twin A and twin B. VAF was calculated by aggregating reads from all normal samples, excluding the spleen sample from twin A and the skin sample from twin B due to cross-contamination of those samples with material from the other twin. The measure of centre is the observed VAF (mutant reads divided by total reads at a locus). Error bars show 95% confidence intervals derived from the binomial distribution based on the sequencing depth. **D** Presents the study design to sample the placenta. **E** Compares the VAFs of early embryonic mutations in each of the bulk and LCM placental samples. **F** Presents the reconstructed phylogeny, which shows a possible and congruent record of the early events associated with twinning. The bar charts represent the lineage composition at each point on the phylogeny, with each bar representing the estimated fraction of cells that belong to each of the tree lineages. Error bars show 95% confidence intervals derived from the binomial distribution based on the aggregated sequencing depth of all mutations associated with each lineage. Source data underlying Fig. 2C, E, F are provided as Source Data files.

We first examined the phylogeny of monozygotic twinning, which, in principle, can assume one of three configurations (Fig. 2A, B). In our data, we identified twenty mutations, which we have categorized into three groups (Fig. 2C and Supplementary Data 3). The first (Fig. 2C, mutation A) was present in both twins. The second (Fig. 2C, mutations B-H) was composed of mutations that made a major contribution to twin A. Some of these mutations approached a variant allele fraction (VAF) of 0.5 in twin A, indicating that the cell in which they arose gave rise to nigh all the cells of this twin. The third (Fig. 2C, mutations I-T) group consisted of variants which were restricted to the tissues of twin B. Next, we assessed the contribution of twin lineages to the placenta, sampled systematically across six different territories (*n* = 18 samples; Fig. 2D, E and Supplementary Fig. 2). Unexpectedly, we found a dominant contribution of twin B’s lineages to placental samples.

From the distribution of developmental mutations across tissues, we derived a phylogeny of twinning (Fig. 2F), which illustrated the asymmetric fate of the earliest lineages: while one lineage (marked by mutations C and F in Fig. 2F) contributed almost exclusively to twin A, another (marked by mutation A in Fig. 2F) contributed to both twins and the placenta, and the third (marked by mutations J,L,Q,T,I,N,P in Fig. 2F) contributed to twin B and the placenta (Supplementary Figs. 2, 3 and Supplementary Note 2). The sum of VAFs across the three parallel lineages was 0.5 in each twin, indicating that we have captured all early lineages that contributed to sampled tissues in both twins (Supplementary Fig. 4).

### Tumour origin and spread

**Fig. 3:**
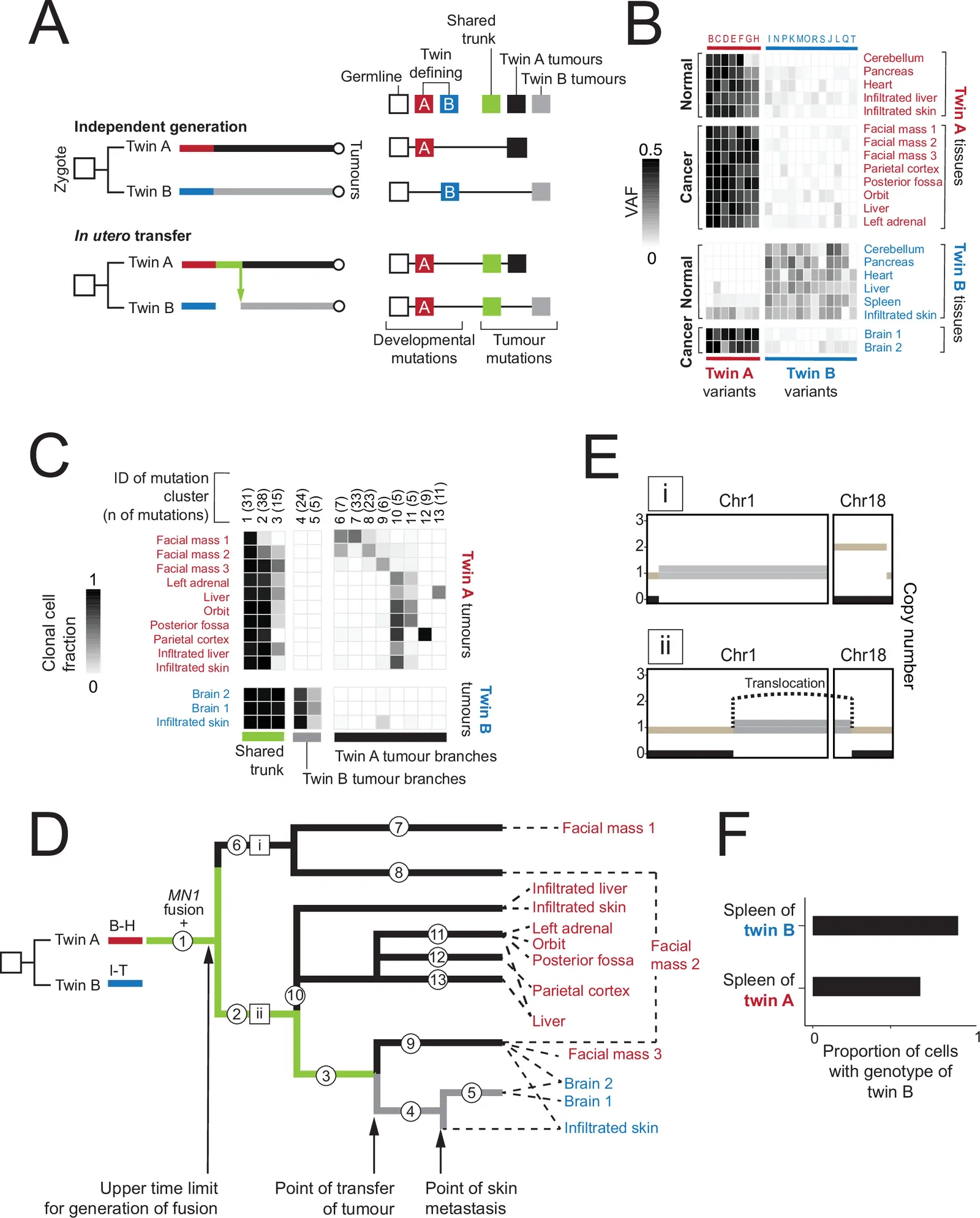
Evidence for tumour transmission and transfusion between twins. **A** Represents possible explanations for the presence of the tumour in both twins and the expected pattern of mutation sharing in the tumour in each case. **B** Shows VAFs of each early embryonic mutation (A-T) genotyped in normal samples of twins and tumour samples from twin A and twin B. **C** Shows the clonal cell fractions of mutation clusters 1–13 in each tumour sample. **D** Shows the configuration of chromosome 1 and 18 copy number changes in two parallel clones of the tumour. The rearrangement between chromosomes 1 and 18 is indicated with a curved line. Phased chromosomes are indicated in beige and black. Segments of the chromosome that could not be phased are indicated in grey. **E** Presents the reconstructed phylogeny of the tumour, with the shared trunk highlighted in green. i, ii Refer to the copy number changes shown in (**D**). **F** Presents the estimates of the fraction of cells from twin B in the spleen of each twin. Source data underlying Fig. 3B, C, F are provided as Source Data files.

We next reconstructed the origin of the sarcoma, distinguishing between two scenarios (Fig. 3A): two independent tumours, or the origin of a tumour in one twin followed by transfer to the other. We exclusively found the genotype of twin A in tumour tissues, irrespective of whether they were sampled in twin A or in twin B, which establishes that the cancer formed in twin A and then transferred to twin B (Fig. 3B and Supplementary Fig. 5). The tumour derived from the cellular lineage with mutations B-H.

We reconstructed the subclonal composition of the tumour using patterns of shared mutations and copy number alterations (Fig. 3C–E and Supplementary Figs. 6, 7). We find that tumour dissemination in twin A likely represented the spread of multiple clones, whereas the tumours of twin B likely spread only once and was a relatively late event (Fig. 3E, Supplementary Fig. 8 and Supplementary Note 3). Assuming a linear mutation rate^12^, we can time the origin of the tumour as occurring at the end of the first trimester, and the spread between twins as occurring late in the second trimester (Supplementary Figs. 9, 10 and Supplementary Note 4).

In assessing all tissues for twin-defining mutations, we found mutations of twin B, the tumour recipient twin, in the solid splenic tissue sample of twin A (tumour donor twin) (Fig. 3F and Supplementary Fig. 11). These mutations were at remarkably high VAFs, consistent with 75% of the cells in the spleen of twin A actually deriving from twin B. These observations are consistent with a mixing of blood cells between the twins, with flow from twin B to twin A.

## Discussion

Our phylogenetic reconstruction provides a detailed account of twinning and establishes the key events associated with cancer formation and spread between twins. We can unambiguously state that the cancer arose from cells of twin A and transferred to twin B. Based on both clinical and genetic observations, the most likely site of tumour origin was the facial mass in twin A (Supplementary Note 1). After early diversification of the tumour and multiple metastases within twin A, the tumour’s spread to twin B occurred late and, most likely, only once. Taken together, these observations suggest that the spread of the tumour was a rare event and therefore not a foregone conclusion. Our analysis resolves the clonal architecture of twinning and the contribution of the early cell lineages to both twins and the placenta in this case. Whilst there have been previous efforts to study twinning from post-zygotic mutations^13^, the clarity afforded by deep whole-genome sequencing of multiple somatic and placental tissues in our investigation revealed marked lineage asymmetries in early embryogenesis. Both twins were mainly formed from separate early lineages, with only one mutation found in both twins. Remarkably, with the caveat that we could not sample the entire organ, the placenta was mostly derived from cells that contributed to twin B.

Despite long-standing interest, our understanding of the early events associated with monozygotic twinning is limited. Under a model of twinning proposed by Corner^14^, monochorionic diamniotic twins such as these result from splitting of the embryo after the formation of the trophectoderm but before the formation of the amnion. This model suggests that the split takes place at around day 6 post conception, when the embryo is composed of ~100 cells^15^. In an alternative theory of twinning^16^, monochorionic diamniotic twins separate at the first cleavage division of the zygote, grow as separate blastocysts within the zona pellucida, and their trophectoderm fuses within the zona pellucida prior to implantation. This model predicts that each twin would have largely its own set of mutations, while the fusion of trophectoderm makes it possible for the trophectoderm of one twin to replace that of the other.

The timing proposed by the classical model does not explain twinning well in our case unless special conditions are met. Let us imagine that one daughter of the zygote is labelled red and the other blue, and that each descendant of those daughters preserves that colour. With even a little mixing of cells during blastulation, or a random orientation of the fission, it is unlikely that cleaving the blastocyst in two at the hundred cell stage would result in one fully red twin and one fully blue twin (Supplementary Fig. 12A). This, however, is essentially what we have observed, with, after a single shared mutation, each twin bearing its own set of mutation ‘labels’. Our data are more compatible with an earlier split, although at least four cells must have existed at the time of the split for mutation A to be present in the soma of both twins (Twin A must have at least one cell with mutation A and at least one cell with mutations CF, while Twin B must have at least one cell with mutation A and one cell whose daughters would acquire mutations JLQTINP). Trophoblast fusion (invoked by the early split model) would explain the distribution of twin lineages in the placenta we observe here, with a dominant contribution to the placenta from twin B. With the caveat that our analysis is limited to surviving cells (Supplementary Fig. 12B), and that we have observed only one instance of twinning and other arrangements may be found^17^, our data argue for an earlier split of the embryo into the two monozygotic twins.

Overall, as well as mapping tumour spread *in utero*, our work delineates a powerful approach to reconstructing the earliest steps that lead from the zygote to the genetic segregation of twins.

## Methods

### Ethics statement

The children’s parent provided written informed consent for participation in our research study, which has been approved by a National Health Service Research Ethics Committee (reference 16/EE/0394), and publication of the associated data.

### Statistics and reproducibility

No statistical method was used to predetermine sample size. No data were excluded from the analyses.

### DNA extraction and sequencing

Bulk DNA was extracted from fresh-frozen tissues using AllPrep DNA/RNA minikit (Qiagen 80204). Placental samples were fixed in formalin and embedded in paraffin. They were extracted using the Qiagen QIAamp DNA FFPE Tissue Kit according to the manufacturer’s instructions. Short-insert genomic libraries were constructed, and 150 base pair paired-end sequencing reads were generated on the Illumina HiSeq X or NovaSeq platforms according to Illumina no-PCR library protocols. Sections of the placental tissue were prepared for LCM according to a previously described protocol^18^. Sample metrics are shown in Supplementary Data 1.

### RNA extraction and sequencing

RNA extraction and sequencing was performed as previously described^19^. Samples for RNA extraction were frozen and stored at −80 °C. RNA extraction was performed using standard methods with the QIAmp RNeasy kit.

### DNA sequencing alignment

All DNA sequences were aligned to the GRCh38 reference genome using the Burrows–Wheeler (BWA-MEM) algorithm^20^.

### RNA sequencing alignment

RNA sequences were aligned to the GRCh37 reference genome and aligned using STAR^21^. Reads that identify the MN1-ZNF341 fusion are shown in Supplementary Data 2.

### Somatic mutation calling

We called mutations from different classes using the pipeline established at the Wellcome Sanger Institute. Substitutions were called with the CaVEMan algorithm^22^ against an in silico-generated normal GRCh38 reference genome. We removed variants with reads with median mapping quality below 140 (ASMD > = 140) and required that more than half of the reads supporting variants were not clipped (CLPM = 0). For all reads, we set a cut-off base quality (25) and mapping quality (30). We generated a pileup from all.bam files. Small insertions and deletions (indels) were identified using the Pindel algorithm^23^. Chromosomal rearrangements were identified with the BRASS algorithm (https://github.com/cancerit/BRASS)^24^ (Supplementary Data 4). Copy number alterations were identified using ASCAT^25^ (Supplementary Data 5).

### Somatic mutation filtering

We employed a series of filters to remove likely artefactual mutations, and required a mutation to fulfil the following criteria to call it somatic:

- Position mapped to chromosomes 1–22 or chromosome X (not chromosome Y).
- No indels present within 30 base pairs of the substitution.
- Minimum 300 reads spanning the position across normal samples from twin A and twin B.
- Maximum 600 reads spanning the position across normal samples from twin A and twin B.
- Presence of minimum 4 variant-reporting reads and VAF minimum 0.1 in at least one sample.
- No significant strand bias observed in reads from all samples from twin A and twin B (normal and tumour), tested with Fisher’s exact test (alpha 0.05, corrected for multiple testing using Bonferroni correction).
- Ratio of the number of reads identified in high-quality (mapping quality 30) to low-quality (mapping quality 5) minimum 0.7 across all samples from twin A and twin B (normal and tumour). This filter removes variants on poorly mapped reads, which could represent artefacts and for which we may not be able to reliably estimate the VAF.

Mutations retained and excluded after each filter are shown in Supplementary Fig. 12.

### Germline mutation filtering

To remove germline variants, we perform a one-sided exact binomial test to identify variants which are heterozygous in all cells (VAF > 0.5), as described in Lawson et al.^26^. Briefly, for each twin, we aggregated reads across all normal samples and obtained the VAF of each mutation by dividing the number of variant-reporting reads by the total number of reads and tested if this mutation is likely to be germline (VAF > 0.5) in this twin (at alpha 0.01). To be excluded, the mutation has to be classified as germline in both twins. *P*-values were corrected for multiple testing with Bonferroni correction. One thousand and sixty-nine mutations were retained after excluding artefacts and likely germline mutations identified in this way. In this set of mutations, we identified 617 mutations present in all normal samples and, based on their VAF ~0.3 and elevated coverage, found that those are more likely variants associated with copy number-altered regions in the germline (Supplementary Fig. 13). We excluded those variants by excluding mutations with coverage (aggregated over all normal samples) above 1.25 or below 0.75 median of the depth of all mutations present on the chromosome, and mutations which were present in clustered (defined as 10 mutations of the 1069 being present within a region of 50 kb). After excluding sequencing artefacts and likely germline mutations, we obtained a set of 542 mutations. We manually reviewed all of these mutations on JBrowse^27^, checking for the mutation occurrence in both read orientations, at different read positions and presence on reads that with high sequencing and mapping quality and were not affected by the presence of a nearby insertion or deletion that could lead to mis-estimation of the mutation VAF. In addition, we excluded variants which, following this filtering process, could not be excluded to be germline. Following manual quality control, we retained 254 mutations that were used to reconstruct the phylogeny. Mutations identified as germline were removed.

### Genotyping of placental samples

Substitutions in LCM placental samples were called with the CaVEMan algorithm^23^ against an in silico-generated normal GRCh38 reference genome. We required cut-off base quality (30) and mapping quality (30). We generated a pileup from all.bam files using the cpgVAF function, searching for the 20 early embryonic mutations and 212 tumour mutations. We then calculated the VAF for each of the mutations classified as early embryonic in each of the trophoblast samples, as well as all bulk placental samples aggregated together. Early embryonic and tumour mutations were genotyped in bulk placental samples using bcftools mpileup^28^ with cut-off base quality (30) and mapping quality (30).

### Estimation of tumour cell fraction in normal samples

Sharing of somatic mutations between tumour and normal samples could result from their shared developmental origin or infiltration of normal samples with tumour cells. To identify tumour-infiltrated samples, we used two approaches based on the presence of tumour-specific mutations and evidence of copy number alterations in normal samples. Results from both approaches were congruent, with estimates from copy number alterations being more sensitive to low-level tumour infiltration.

First, purity can be estimated by taking advantage of the loss of chromosomes 1q and 18p in tumour samples. Under the assumption that those alterations are specific to tumour cells and no copy number changes are present in normal samples, one would expect the VAFs of heterozygous SNPs and germline mutations in those regions to be at 0.5, while in a tissue sample which is infiltrated by tumour cells, we expect a systematic deviation from 0.5. We phased heterozygous SNPs on chr1q and chr18p; all SNVs in the copy-number altered regions which were found at VAF > 0.75 in the posterior fossa tumour sample (purest tumour sample available) were assigned to one chromosome, while all SNVs present at VAF < 0.25 were assigned to the other chromosome. In each normal sample, we calculated the median VAF of mutations found on either chromosome and derived the expected fraction of cells which lost the allele (i.e. tumour cells). Purity estimates based on mutations on chr1q and chr18p were consistent for each sample. To obtain the final estimate of purity, we averaged the estimates based on chr1q SNPs and chr18p SNPs. The deviation from the expected value of 0.5 was consistent across the entire lost segment in both chr1q and chr18p for each sample (Supplementary Fig. 14), arguing against focal copy number alterations in normal samples that would have confounded our estimates. Estimates are shown in Supplementary Data 6.

To validate our estimates of purity, we also estimated tumour infiltration in normal samples by determining the fraction of reads reporting clonal tumour mutations in normal samples (Supplementary Fig. 15). First, we identified 168 mutations present in all tumour samples and with VAF (aggregated across all tumour samples) min 0.2, and plotted VAF in the tumour against VAF of these mutations in each normal sample. For samples with no tumour infiltration, we expect to identify a cluster of mutations at VAF > 0.2 in the tumour and VAF = 0 in the normal sample. If the normal sample is heavily tumour-infiltrated, we expect that no separate cluster of mutations at VAF = 0 in normal will be present. With this line of reasoning, we identified highly pure samples and selected mutations which were present at VAF > 0.2 in the tumour, absent from highly pure samples, and at VAF < 0.1 in aggregated twin A samples to exclude potential embryonic mutations.

The observed VAF of a clonal tumour mutation each sample is given as

$${V}_{o}={f}_{t}*{V}_{t}+(1-{f}_{t})*{V}_{n}$$

where:

- *V*_*o*_: VAF observed in the sample
- *V*_*t*_: VAF of the mutation in tumour cells
- *V*_*n*_: VAF of the mutation in normal cells
- *f*_*t*_: fraction of tumour cells
- (1 − *f*_*t*_): fraction of normal cells (1 − *f*_*t*_)

As we searched for mutations that are clonal in the tumour and absent in normal samples, we assume *V*_*t*_ to be 0.5 and *V*_*n*_ to be 0. The tumour cell fraction is given as

*f*_*t*_ = *V*_*o*_ / *V*_*t*_

Estimates are shown in Supplementary Data 7.

### Classification of somatic mutations and reconstruction of normal phylogeny

We categorized each of the mutations present in the set of 254 high-confidence mutations as belonging to one of the following categories:

- Present in normal samples from both twins
- Present in normal samples in twin A only
- Present in normal samples in twin B only
- Specific to the tumour (allowing for presence in tumour-infiltrated normal samples)
- Present in a single normal sample
- Present in a single tumour sample

A phylogeny was then constructed manually according to the pattern of mutation presence and VAF across samples. We also considered which trophoblast samples each normal mutation was confidently identified in. Assignment of mutations to the phylogeny is shown in Supplementary Data 8. Coordinates of 20 early embryonic mutations used to reconstruct the early events associated with twinning (labelled A-T) are shown in Supplementary Data 3. VAFs of the 20 early embryonic mutations used to reconstruct twinning in each sample are given in Supplementary Data 9. VAFs of each of the tumour mutations are given in Supplementary Data 10.

### Reconstruction of tumour phylogeny

We manually clustered 212 tumour-specific mutations into 13 clusters, each containing at least 5 mutations, based on the following criteria:

- Cluster 1: mutations present in all tumour samples, VAF > 0.1 in the facial mass (sample 1)
- Cluster 2: mutations present in min 7 tumour samples, absent from the facial mass (sample 1)
- Cluster 3: mutations found in tumour samples from twin B, facial mass (samples 2 and 3), brain orbit, posterior fossa, left adrenal and liver tumour samples
- Cluster 4: mutations identified only in tumour samples from twin B, VAF > 0.1 in both samples
- Cluster 5: mutations identified only in tumour samples from twin B, VAF > 0.1 in one sample
- Cluster 6: mutations found at VAF > 0.1 only in facial mass (samples 1 and 2)
- Cluster 7: mutations found at VAF > 0.1 only in facial mass (sample 1) tumour
- Cluster 8: mutations found at VAF > 0.1 only in facial mass (sample 2) tumour
- Cluster 9: mutations found at VAF > 0.1 only in facial mass (sample 3) tumour
- Cluster 10: mutations found at VAF > 0.1 in right parietal, brain orbit, posterior fossa, left adrenal and liver tumour samples
- Cluster 11: mutations found at VAF > 0.1 in brain orbit, posterior fossa, left adrenal and liver tumour samples, but not in the right parietal tumour
- Cluster 12: mutations found at VAF > 0.1 only in right parietal tumour sample
- Cluster 13: mutations found at VAF > 0.1 only in liver tumour sample

We reconstructed the phylogeny of the tumour based on VAFs of mutations of each cluster in each sample, following the pigeonhole principle^23^. Assignment of each tumour mutation to a cluster is provided in Supplementary Data 11. Mean VAFs of each cluster in each sample are given in Supplementary Data 12. Tumour clonal composition (cancer cell fraction of each cluster; calculated as the CCF of the parent − sum of CCF of all its direct children) is given in Supplementary Data 13.

### Estimation of cell transfer between twins

We estimate the levels of twin-to-twin transfusion using VAF values of twin A- and twin B-specific mutations. The observed VAF of a mutation in twin A spleen is given as

$${V}_{o}={f}_{{twinA}}*{V}_{{twinA}}+{f}_{{twinB}}*{V}_{{twinB}}$$

where:

- *V*_*o*_: VAF observed in the spleen sample
- *V*_*twin A*_: VAF of the mutation in twin A (non-spleen)
- *V*_*twin B*_: VAF of the mutation in twin B (non-spleen)
- *f*_*twin A*_: fraction of twin A cells in twin A spleen
- *f*_*twin B*_: fraction of twin B cells in twin A spleen

For the 7 twin A-specific mutations, we estimate twin A VAF using non-spleen normal samples and assume VAF in twin B to be 0. We can calculate *f*_*twin* A_ as

*f*_*twin A*_ = *V*_*o*_ /*V*_*twin A*_

and *f*_*twin* B_

*f*_*twin B*_ = *V*_*o*_ /*V*_*twin B*_

Estimates are provided in Supplementary Data 14.

### Magnetic resonance imaging

The images in Fig. 1 were obtained from standard clinical MRI sequences. The MRI scans were performed on a Siemens 1.5 T Avanto Fit (Siemens Erlangen, Germany). Whole body imaging was acquired in multiple sections in the coronal plane. T2 BLADE sequences were acquired in the coronal section (slice thickness 3 mm, flip angle 120°, TR 4461, TE 89, number of averages 1, ETL 41, slice spacing 3.3 mm). Echoplanar 2D diffusion-weighted imaging was acquired through thorax, abdomen and pelvis in the axial plane (*B* values 0, 100, 500 and 100; ST 6 mm, TR 4800, TE 55, number of averages 1, slice spacing 7.2 mm, ETL 39, flip angle 90). Head images T2 DESTIR sequences were acquired in the axial and coronal planes (ST 4 mm, TR 8340, TE 113, averages 1, Inversion time 130, ETL 10). Volumetric gradient echo T1 was acquired for multiplanar reconstruction (ST 1 mm, TR11, TE 4.94, Averages 1, ETL 1, flip angle 15). Fluid-attenuated T2 was acquired in the coronal plane (ST4, TR 9000, TE 108, IR 2500, ETL20, flip angle 150). Susceptibility imaging was acquired in the axial plane (T2 SWI TRA, ST 2.5, TR 49, TE 40, Averages 1, ETL 1). Diffusion imaging was acquired in the axial plane at B0 and 1000 (ST 4 mm, TR 5950, TE 87, Averages 1, ETL 0).

### Reporting summary

Further information on research design is available in the Nature Portfolio Reporting Summary linked to this article.

## Supplementary information

Supplementary Information

Description of Additional Supplementary Files

Supplementary Dataset

Reporting Summary

Transparent Peer Review File

## Source data

Source Data

## Data Availability

All sequencing data have been deposited in the European Genome-phenome Archive (accession number EGAS00001007536; hyperlink: https://ega-archive.org/studies/EGAS00001007536). This is a permanent repository. The raw data are available under restricted access due to data privacy laws. Access may be granted following an application to the Data Access Committee (datasharing@sanger.ac.uk). A response is usually provided within 3 weeks. Once a request is approved, raw data may be downloaded and used for the duration of time agreed with the Data Access Committee. Source data required to reproduce each figure, processed data and somatic mutation calls are provided with this manuscript. Source data are provided with this paper.

## Code availability

Custom code developed for the purpose of this analysis has been deposited on Zenodo^29^ and is available on GitHub under the link: https://github.com/BehjatiLab/tumour_transmission_mz_twins.
