# Supplementary material for: Embryonic origin of cancer in newborn twins
Source: Nat Commun. 2026 Oct 6;17:11246. doi: 10.1038/s41467-026-78178-5 (PMC13723494; doi:10.1038/s41467-026-78178-5)
Supplement: Supplementary file 1 [file 41467_2026_78178_MOESM2_ESM.pdf]

Description of Additional Supplementary Files

Supplementary Data 1 – sample overview

Supplementary Data 1 – MN1-ZNF341 fusion reconstruction

Supplementary Data 3 – coordinates and classification of 20 early embryonic mutations

Supplementary Data 4 – BRASS calls

Supplementary Data 5 – ASCAT calls

Supplementary Data 6 – purity from CN Sam Behjati FMedSci Chair / Professor of Paediatrics Director, Cambridge Children's Research Institute Honorary Consultant Paediatric Oncologist Department of Paediatrics, University of Cambridge Box 116, Level 8, Cambridge Biomedical Campus Cambridge CB2 0QQ Phone: +44 (0) 1223 69005 E-mail: sb849@cam.ac.uk

Supplementary Data 7 – purity from SNVs

Supplementary Data 8 – assignment of 254 somatic mutations to phylogeny

Supplementary Data 9 – VAFs of 20 early embryonic mutations in each sample

Supplementary Data 10 – VAFs of 212 tumour mutations in each sample

Supplementary Data 11 – assignment of tumour mutations to clusters 1-13

Supplementary Data 12 – VAFs of tumour mutation clusters 1-13 in each sample

Supplementary Data 13 – clonal tumour composition

Supplementary Data 14 – estimates of cell transfer between twins
