# Supplementary material for: Embryonic origin of cancer in newborn twins
Source: Nat Commun. 2026 Oct 6;17:11246. doi: 10.1038/s41467-026-78178-5 (PMC13723494; doi:10.1038/s41467-026-78178-5)
Supplement: Supplementary file 3 [file 41467_2026_78178_MOESM5_ESM.pdf]

nature portfolio

Peer Review File

Embryonic origin of cancer in newborn twins

Corresponding Author: Professor Sam Behjati

This file contains all reviewer reports in order by version, followed by all author rebuttals in order by version.

Version 0:

Reviewer comments:

Reviewer #1

(Remarks to the Author)
This paper provides a detailed study of early twinning and tumor formation. I enjoyed reading the paper very much. It's a unique dataset, and the paper presents a very principled approach to dissecting tumor initiation. I have a few minor comments, mainly about the methods and the phylogeny.

1. It might be good to determine the GL genotype of the tumor in terms of the 20 mutations. Do you believe the tumor to be descendant from normal cells with mutations G,H (and thus also mutations C,F,B,D,E)? It looks like this might be the case judging from Fig 3B.

2. For tumor SNVs, please show CCFs rather than VAFs (as done in Fig 3F). That is, please correct for purity and any copy number aberrations. It will make the figures easier to understand (such as Fig 3c, Supp Fig 5, Supp Fig 6, etc.). Relatedly, it'd be great if the manual phylogeny reconstruction was done using CCFs rather than VAFs. Given the mostly high purity values, I do not expect results to change, but it'd be still good to verify.

3. Some additional comments about the phylogeny presented in Fig 3E:

* It is unclear why clusters 11, 12 and 13 are viewed as siblings of a shared parent defined by no mutations. If there were no common mutations (distinct from clusters 1, 2 and 10), why aren't 11, 12 and 13 direct children of 10?

* Is the reason for placing the MN1 fusion on the trunk of the tree that it was observed in each tumor sample?

* Would it be possible to include the CNA events (such as those listed in Fig 3D) on the phylogeny?

* Also, please include a supplementary table showing the presence/absence (or even better, proportion) of each tumor clone in each sample? (Proportion of a clone in a sample would be CCF of the defining cluster minus the sum of CCFs of its children.)

4. For Supp. Fig 9, please estimate signature exposures (e.g. using SigProfiler). It'd be great if SBS1 and SBS5 indeed correspond to the majority of exposures (rather than providing solely a visual argument).

Minor:
- line 314: add "normal" in front of sample

(Remarks on code availability)

Reviewer #2

(Remarks to the Author)
This manuscript describes a very rare, unusual situation as far as the sarcoma development - It is interesting that they were able to show an initial transfer of sarcoma cells from twin A to B, and then apparently some of those tumor cells transferred the opposite direction at a later point. In a way it’s not surprising - the tumor cells had metastatic potential and two hosts

being exposed to the cells, so that sort of back and forth would be expected. The relative timing of the tumor origin and passage between twins is fascinating but follows expected behavior of metastatic disease and likely also the potential of hematopoietic stem cells that might follow similar twin-twin trajectories. The data analysis is remarkably thorough and provides a satisfying and complete assessment. The “extended data” and “supplementary data” sections along with the main data figures provide a somewhat confusing array of sections for a reader to cross-reference to try and take in all the information, but perhaps that was arranged that way on instruction from the journal.

The authors take advantage of the tissue to explore clonal movements between the twins in the twinning process itself, and this is the more interesting part of the manuscript. This careful lineage tracking was afforded by the deep sequencing and careful tracking of allelic fraction, and the implications of this clonal mutation tracking is not discussed with the known timing of monozygotic twinning or longstanding hypotheses (Corner’s). Does this information inform or alter our understanding of the monozygotic twinning process? Where does the information gleaned in the sequencing analysis place this particular twin within known paradigms?

Experiments are carefully performed and analyzed and conclusions suitably, but the figures are hard to understand without referral to the text. This is okay for the main figures, but the extended figures need more annotation. Labels for the mutations A to T for a start. While these are also featured in Figure 2, there is no listing in the manuscript or reference to Table S8 where these reside.

“cells in the spleen” that were sequenced appear to be blood cells, but this is not clear. More understanding of the prep of the spleen cells might help – is any solid spleen tissue a part of this? White or red pulp?

On this same comment, the “mixing” of cells was not considered in analysis of other tissues, but only spleen and skin. Unclear why blood contamination was not an issue for other tissues?

Extended data 4 figure: unknown what the acronyms refer to.

(Remarks on code availability)
I haven't run any code. I believe that the analysis was performed with competence.

Version 1:

Reviewer comments:

Reviewer #1

(Remarks to the Author)
Thanks for the careful revision --- my comments have been addressed. One small follow-up on the CCF point: thanks for moving Fig 3C and Table S13 to CCF, but Supp Figs 5–7 and Table S12 are still VAF-based, as are the cluster definitions and phylogeny reconstruction in the methods ("VAF > 0.1"); it'd be good to re-plot these in CCF for consistency.

(Remarks on code availability)

Reviewer #2

(Remarks to the Author)
Thank you for the clarifications of figures, discussion of twinning processes, and other changes that were in response to the reviewer comments. No further comments.

(Remarks on code availability)

Open Access This Peer Review File is licensed under a Creative Commons Attribution 4.0 International License, which permits use, sharing, adaptation, distribution and reproduction in any medium or format, as long as you give appropriate credit to the original author(s) and the source, provide a link to the Creative Commons license, and indicate if changes were made.
In cases where reviewers are anonymous, credit should be given to 'Anonymous Referee' and the source.
The images or other third party material in this Peer Review File are included in the article's Creative Commons license, unless indicated otherwise in a credit line to the material. If material is not included in the article's Creative Commons license and your intended use is not permitted by statutory regulation or exceeds the permitted use, you will need to obtain permission directly from the copyright holder.
To view a copy of this license, visit https://creativecommons.org/licenses/by/4.0/

Rebuttal for manuscript 'Embryonic origin of cancer in newborn twins' NCOMMS-25-100929A

We thank the Editor and Reviewers for their most helpful comments which we have addressed as detailed below. The page and line numbers refer to the revised manuscript

Referee 1

#	Comment	Reply
1.1	This paper provides a detailed study of early twinning and tumor formation. I enjoyed reading the paper very much. It's a unique dataset, and the paper presents a very principled approach to dissecting tumor initiation. I have a few minor comments, mainly about the methods and the phylogeny.	We thank the reviewer for their comments about our work.
1.2	It might be good to determine the GL genotype of the tumor in terms of the 20 mutations. Do you believe the tumor to be descendant from normal cells with mutations G,H (and thus also mutations C,F,B,D,E)? It looks like this might be the case judging from Fig 3B.	We thank the reviewer for this comment. We concluded that the tumour descended from the lineage that had all of mutations B-H since, as the reviewer points out, all these mutations are present (and are clonal) in the tumour.<br><br>**Changes to manuscript:**<br>1) We now indicate these mutations on the tumour phylogeny in Fig. 3E<br>2) We have added the sentence 'The tumour derived from the cellular lineage with mutations B-H.' to line 86.
1.3	For tumor SNVs, please show CCFs rather than VAFs (as done in Fig 3F). That is, please correct for purity and any copy number aberrations. It will make the figures easier to understand (such as Fig 3c, Supp Fig 5, Supp Fig 6, etc.). Relatedly, it'd be great if the manual phylogeny reconstruction was done using CCFs rather than VAFs. Given	We have now calculated CCF using the values of VAF and tumour purity as described in Tarabichi et al., 2021[1]. We have also re-analysed the phylogeny using the CCF rather than the VAF values, with, as the reviewer surmised, minimal changes. We discuss the reconstructed phylogeny in reply to the comment below.<br><br>**Changes to manuscript**:

	the mostly high purity values, I do not expect results to change, but it'd be still good to verify.	1) We have re-created the figures indicated (Fig. 3c, Supp Fig 5 and Supp Fig 6). These figures have now been updated in the main figures and supplementary material.
1.4	Some additional comments about the phylogeny presented in Fig 3E: It is unclear why clusters 11, 12 and 13 are viewed as siblings of a shared parent defined by no mutations. If there were no common mutations (distinct from clusters 1, 2 and 10), why aren't 11, 12 and 13 direct children of 10?	Thank you for drawing this to our attention. We realise that the phylogeny was not presented clearly. Clusters 11 and 12 are, in fact, the direct descendants of 10, and we have altered the phylogeny accordingly. In reviewing the phylogeny during the course of the revision, we have realised that although cluster 13 could be the descendant of either clusters 10 or 3, it is more likely that it is the descendant of 3, and we have therefore placed cluster 13 here.<br><br>**Changes to manuscript**:<br>1) We have corrected the phylogeny as shown on Fig. 3E.
1.5	Is the reason for placing the MN1 fusion on the trunk of the tree that it was observed in each tumor sample?	Yes, the *MN1*::*ZNF341* fusion was observed in all tumour samples which we have emphasised in this revision:<br><br>**Changes to manuscript:**<br>1) We added to the main text this sentence: “Biopsies of their tumours were reported as *MNF1*::*ZNF341* rearranged undifferentiated sarcomas, with all tumour samples from both children bearing this driver fusion” (line 46).<br>2) We discuss this in the supplementary note.
1.6	Would it be possible to include the CNA events (such as those listed in Fig 3D) on the phylogeny?	The CNA events are indicated on the phylogeny on Fig. 3E as squares (i) and (ii), referring to CN changes shown on Fig. 3D. We now amended the legend of the Figure 3D to clarify that those annotations refer to the copy number alterations.<br><br>**Changes to the manuscript:**<br>1) We have amended the legend for Figure 3: **“**Panel E presents the reconstructed phylogeny of the tumour, with the shared trunk highlighted in green. i) and ii) refer to the copy number changes shown on Panel D. “ (lines 239-240)

1.7	Also, please include a supplementary table showing the presence/absence (or even better, proportion) of each tumor clone in each sample? (Proportion of a clone in a sample would be CCF of the defining cluster minus the sum of CCFs of its children.)	We now provide these values in supplementary table 13.<br><br>We note that there are two instances where the sum of the CCFs of the children exceeds that of the parent cluster. We believe that this is most likely due to the low number of mutations in some of these clusters and the limited coverage over some of these mutations, which may lead to mean VAF and CCF estimates of each cluster being imprecise.
1.8	For Supp. Fig 9, please estimate signature exposures (e.g. using SigProfiler). It'd be great if SBS1 and SBS5 indeed correspond to the majority of exposures (rather than providing solely a visual argument).	We thank the reviewer for this suggestion. We have performed signature attribution using SigProfiler Attribution[2] to COSMIC signatures (version 3.5) The mutations in the tumour are decomposed into signatures SBS1, SBS5, and SBS40a. All of SBS1, SBS5 and SBS40a are known to be clock-like signatures that accumulate linearly with age in normal tissues[3].<br><br>**Changes to manuscript**:<br>1) We have now added the signature attribution from SigProfiler to Supplementary Figure 9 (panel B).
1.9	Minor:<br>- line 314: add "normal" in front of sample	We thank the reviewer for the comment. We have now amended this.

Referee 2 –

#	Comment	Reply
2.1	This manuscript describes a very rare, unusual situation as far as the sarcoma development - It is interesting that they were able to show an initial transfer of sarcoma cells from twin A to B, and then apparently some of those tumor cells transferred the opposite direction at a later point. In a way it's not surprising - the tumor cells had metastatic potential and two hosts being exposed to the cells, so that sort of back and forth would be expected. The relative timing of the tumor origin and passage between twins is fascinating but	We thank the reviewers for their helpful and supportive comments. The tumour indeed spread from twin A to twin B, and we are able to time this event. Blood cells spread back from twin B to twin A, as the reviewer says.<br><br>We apologize for the confusion caused by the arrangement of the additional material. To help readers orient themselves in the text, we moved all extended data figures to the supplementary material.

	follows expected behavior of metastatic disease and likely also the potential of hematopoietic stem cells that might follow similar twin-twin trajectories. The data analysis is remarkably thorough and provides a satisfying and complete assessment. The "extended data" and "supplementary data" sections along with the main data figures provide a somewhat confusing array of sections for a reader to cross-reference to try and take in all the information, but perhaps that was arranged that way on instruction from the journal.	
2.2	The authors take advantage of the tissue to explore clonal movements between the twins in the twinning process itself, and this is the more interesting part of the manuscript. This careful lineage tracking was afforded by the deep sequencing and careful tracking of allelic fraction, and the implications of this clonal mutation tracking is not discussed with the known timing of monozygotic twinning or longstanding hypotheses (Corner's). Does this information inform or alter our understanding of the monozygotic twinning process? Where does the information gleaned in the sequencing analysis place this particular twin within known paradigms?	We thank the reviewer for raising this very interesting point. We now expand on this point in the discussion, and have added Supplementary Figure 12 to support the point visually.<br><br>**Changes to manuscript**:<br><br>1) We now added the following to the discussion (lines 117-141):<br><br>"Despite long-standing interest, our understanding of the early events associated with monozygotic twinning is limited. Under a model of twinning proposed by Corner[4], monochorionic diamniotic twins such as these result from splitting of the embryo after the formation of the trophectoderm but before the formation of the amnion. This model suggests that the split takes place at around day 6 post conception, when the embryo is composed of ~100 cells[5]. In an alternative theory of twinning[6], monochorionic diamniotic twins separate at the first cleavage division of the zygote, grow as separate blastocysts within the zone pellucida, and their trophectoderm fuses within the zona pellucida prior to implantation. This model predicts that each twin would have largely its own set of mutations, while the fusion of trophectoderm makes it possible for the trophectoderm of one twin to replace that of the other.<br><br>"The timing proposed by the classical model does not explain twinning well in our case unless special conditions are met. Let us imagine that one daughter of the zygote is labelled red and the other blue, and that each descendant of those daughters preserves that colour. With even a little mixing of cells during blastulation, or a random

		orientation of the fission, it is unlikely that cleaving the blastocyst in two at the hundred cell stage would result in one fully red twin and one fully blue twin (Extended Data Figure 12A). This, however, is essentially what we have observed, with, after a single shared mutation, each twin bearing its own set of mutation 'labels'. Our data are more compatible with an earlier split. Trophoblast fusion (invoked by the fusion model) would explain the distribution of twin lineages in the placenta we observe here, with a dominant contribution to the placenta from twin B. With the caveat that our analysis is limited to surviving cells (Extended Data Figure 12B), and that we have observed only one instance of twinning and other arrangements may occur[7], our data argues for an earlier split of the embryo into the two monozygotic twins."
2.3	Experiments are carefully performed and analyzed and conclusions suitably, but the figures are hard to understand without referral to the text. This is okay for the main figures, but the extended figures need more annotation. Labels for the mutations A to T for a start. While these are also featured in Figure 2, there is no listing in the manuscript or reference to Table S8 where these reside.	We apologize that this was confusing. We have expanded our figure legends for every supplementary and extended data figure to limit the need to cross-reference.<br><br>**Changes to manuscript**:<br>1) Labels for mutations A to T are now referred to in the main text (line 61-65).<br>2) We have now included an explicit reference to Table S8 (now Table S3) (line 61).
2.4	"cells in the spleen" that were sequenced appear to be blood cells, but this is not clear. More understanding of the prep of the spleen cells might help – is any solid spleen tissue a part of this? White or red pulp?	We thank the reviewer for raising this. The splenic sample was a biopsy of solid spleen, and we have clarified this in the main text by referring to it as 'solid splenic tissue sample'. It will have been composed both of white pulp and red pulp. In all likelihood, the vast majority of cells in this sample will be bone marrow-derived.<br><br>**Changes to manuscript:**<br>1) we found mutations of twin B, the tumour recipient twin, in the **solid splenic tissue sample** of the spleen of twin A (line 96)
2.5	On this same comment, the "mixing" of cells was not considered in analysis of other tissues, but only spleen and skin. Unclear why blood contamination was not an issue for other tissues?	Thank you for bringing up this important point. In our analysis, we have noted: 1) tumour infiltration of the skin in both twins, and 2) contamination of the spleen of twin A with blood from twin B (as evidenced by the presence of the twin B-specific mutations in the spleen of twin A). As both twins were born with a "blueberry muffin"

		type of lesions, that is consistent with the presence of tumour cells in the skin of both twins, including twin B, and explains the presence of twin A-specific mutations in twin B skin sample. The spleen is an organ which stores blood cells and so it is not surprising that blood contamination is most noticeable in this organ, as opposed to other organs (which is consistent with mutations from the opposite twin being absent, or present at a very low VAF, in other normal tissues). We discuss this in detail in the supplementary note 2 "Cell transfer between twins".
2.6	Extended data 4 figure: unknown what the acronyms refer to.	We apologise that this was unclear. We have used the letters to indicate the mutations which were considered to be defining of the different lineages.<br><br>**Changes to manuscript:**<br>1) We have now amended the legend of the Extended Data Figure 4 (now Supplementary Figure 4) to make it clear that we are referring to a set of individual mutations.

References

1. Tarabichi, M. *et al.* A practical guide to cancer subclonal reconstruction from DNA sequencing. *Nat. Methods* **18**, 144–155 (2021).
2. Díaz-Gay, M. *et al.* Assigning mutational signatures to individual samples and individual somatic mutations with SigProfilerAssignment. *Bioinformatics* **39**, btad756 (2023).
3. Wang, Y. *et al.* Systemic mutagen exposures reported by normal kidney cell genomes. 2026.04.07.716715 Preprint at https://doi.org/10.64898/2026.04.07.716715 (2026).
4. Corner, G. W. The observed embryology of human single-ovum twins and other multiple births. *Am. J. Obstet. Gynecol.* **70**, 933–951 (1955).
5. Hardy, K., Handyside, A. H. & Winston, R. M. L. The human blastocyst: cell number, death and allocation during late preimplantation development in vitro. *Development* **107**, 597–604 (1989).
6. Herranz, G. The timing of monozygotic twinning: a criticism of the common model. *Zygote* **23**, 27–40 (2015).

7. Yoon, C. J. *et al.* Clonal dynamics of monozygotic twinning in early human embryogenesis. 2025.10.05.680569 Preprint at https://doi.org/10.1101/2025.10.05.680569 (2025).

Rebuttal for manuscript 'Embryonic origin of cancer in newborn twins' NCOMMS-25-100929-T

We thank the Editor and Reviewers for their most helpful comments which we have addressed as detailed below.

Referee 1

#	Comment	Reply
1.1	Thanks for the careful revision --- my comments have been addressed. One small follow-up on the CCF point: thanks for moving Fig 3C and Table S13 to CCF, but Supp Figs 5–7 and Table S12 are still VAF-based, as are the cluster definitions and phylogeny reconstruction in the methods ("VAF > 0.1"); it'd be good to re-plot these in CCF for consistency.	We thank the reviewer for pointing this out. We have now amended this.

Referee 2

#	Comment	Reply
2.1	Thank you for the clarifications of figures, discussion of twinning processes, and other changes that were in response to the reviewer comments. No further comments.	We thank the reviewer for their interest in the manuscript and contribution to the review process.
