# Supplementary material for: Embryonic origin of cancer in newborn twins
Source: Nat Commun. 2026 Oct 6;17:11246. doi: 10.1038/s41467-026-78178-5 (PMC13723494; doi:10.1038/s41467-026-78178-5)
Supplement: Supplementary file 4 [file 41467_2026_78178_MOESM1_ESM.docx]

**Supplementary Information**

This appendix has been provided by the authors to give readers additional information about their work.

Embryonic origin of cancer in newborn twins

Barbara Walkowiak^1^*, Sadie Baskind^2,3^*, Manas Dave^1,4^, Pooja Balasubramanian^2,3^, Nathaniel Anderson^1^, Jonathan Kennedy^1,2,3^, Thomas R. W. Oliver^1,2,5,6^, Rohan Verma^2,3^, Sarita Depani^2,3^, Simon Hannam^2,3^, Tom Watson^2,3^, Dyanne Rampling^2,3^, Karin Straathof^2,3,6^, Liina Palm^2,3^, J. Ciaran Hutchinson^2,3^, Henry Lee-Six^1,4,5#^, Olga Slater^2,3,#^, Sam Behjati^1,2,3,5,7#^

*Equal contribution

^#^Co-corresponding. Email: [hl11@sanger.ac.uk](mailto:hl11@sanger.ac.uk), [olga.slater@gosh.nhs.uk](mailto:olga.slater@gosh.nhs.uk), [sb31@sanger.ac.uk](mailto:sb31@sanger.ac.uk)

^1^Wellcome Sanger Institute, Hinxton, UK.

^2^Great Ormond Street Hospital NHS Foundation Trust, London, UK.

^3^Great Ormond Street Biomedical Research Centre, London, UK.

^4^Department of Pathology, University of Cambridge, Cambridge, UK.

^5^Cambridge University Hospitals NHS Foundation Trust, Cambridge, UK.

^6^UCL Cancer Institute, London, UK.

^7^Department of Paediatrics, University of Cambridge, Cambridge, UK.

**Table of contents**

Supplementary notes

1. Histology of tumour samples
2. Cell transfer between twins
3. Clonal composition of the tumour in twin A and twin B
4. Timing of tumour origin in twin A and spread to twin B

Supplementary Figures

1. *MN1-ZNF341* fusion reconstruction
2. VAFs of early embryonic mutations in individual normal samples from each twin
3. VAFs of early embryonic mutations in placental samples
4. Estimates of cell lineage contributions to each twin and the placenta
5. VAFs of early embryonic mutations in tumour samples
6. VAFs of tumour mutations associated with cluster 1-13 in each sample
7. Mean VAFs of each tumour mutation cluster 1-13 in each sample
8. Comparison of cluster 1 and 4 in tumour and tumour-infiltrated samples
9. Comparison of tumour mutation burden to other paediatric tumours
10. Trinucleotide contexts of tumour mutations
11. Estimates of transfer of cells in the spleen samples
12. Models of twinning
13. Filtering SNVs
14. Filtering germline SNVs
15. Sample purity estimates from copy number changes
16. Sample purity estimates from SNVs

**SUPPLEMENTARY NOTES**

**Supplementary notes**

17. Histology of tumour samples
18. Cell transfer between twins
19. Clonal composition of the tumour in twin A and twin B
20. Timing of tumour origin in twin A and spread to twin B

**1 Histology of tumour samples**

Skin tumour deposits from both twins were reported as poorly circumscribed subcutaneous nodules composed of small round blue cells with brisk mitotic activity but not other specific morphological features. There was no neurofibrillary stroma and no cellular differentiation. Tumour samples from both twins were reviewed together and were noted to be morphologically identical. Immunostaining for Desmin, Myogenin, CD99, CD45, PHOX2B, and CD117 was negative. CD45, CD68 and CD168 were expressed in a few scattered intralesional cells, likely representing infiltrating T lymphocytes. INI-1 staining/ expression was retained in tumour cells. The neoplastic cells strongly and diffusely expressed CD56. Both tumours were later found to diffusely and strongly express SF1 in keeping with the characteristics published in the MN1 case series^1^.

Taken together, the features of the cancers in both children were of a small round cell tumour most consistent with an undifferentiated sarcoma. They were not consistent with neuroblastoma, leukaemia, mast cell disease, PNET, rhabdoid tumour, Wilms tumour or rhabdomyosarcoma.

**2 Cell transfer between twins**

We note that mutations which we term ‘twin-A specific’ were found at very low VAFs in the spleen and liver samples of twin B, and similarly, mutations called ‘twin B-specific’ were identified at very low VAFs in samples from twin A (**Supplementary Figure 2**). As monozygotic monochorionic twins share circulation *in utero*, those observations can be parsimoniously explained by blood transfer between twins.

Twin B specific mutations were observed at high levels in the spleen of twin A, and at low (VAF < 0.1) levels in the heart, pancreas, liver and skin. No twin B-specific mutations were found in the cerebellum. While we cannot formally exclude this being due to some low-level contribution of early cell lineages to twin A, we find blood transfer from twin B to be the more likely explanation for the following reasons:

1)	The elevation of those mutations in the spleen compared to other tissues argues for this being due to transfusion, especially in this clinical context where twin A presented with active extramedullary haematopoiesis.

2)	There is a moderate positive correlation between the VAF of twin B-specific mutations in twin B and spleen of twin A (Pearsons’ r 0.42), further arguing for this being due to blood transfer.

3)	Very low levels of twin B-specific mutations in other tissues of twin A are consistent with low-level tissue infiltration by blood cells (e.g., immune cells), expected in bulk samples

4)	The estimated contribution of lineages marked by mutations A, and mutations CF, to twin A is 0.999 (**Supplementary Figure 4**), indicating that we have likely captured all early cell lineages which formed twin A and are not missing other lineages forming this twin.

Twin A-specific mutations were observed in the spleen (6 / 7 twin A-specific mutations) and liver of twin B (1 / 7 mutations). The elevation of the VAF of those mutations specifically in the spleen is, again, congruent with this being a result of the sharing of the circulation between twins *in utero*. However, in this case, it is also possible that the sharing is due to the presence of the tumour, as microscopic tumour deposits have been identified in the spleen of twin B.

Given that our estimates suggest blood flow from twin A to twin B only occurred at low levels, it is not surprising that we do not see any evidence of twin A-specific mutations in other tissues.

**3 Clonal composition of the tumour in twin B**

The largest tumour mass in twin A was composed of multiple subclones (**Fig 3C-D, Supplementary Figure 6-7**), and metastases within twin A exhibited contributions from many of them, indicative of polyclonal dissemination. In contrast, the intracranial tumours and skin samples of twin B showed the presence of only two lineages, identified by mutations from clusters 4 and, at low levels, 9 (**Figure 3C, Supplementary Figure 8).** The similarity in clonal composition of these metastatic deposits in twin B suggests that the transfer of cells from twin A to twin B may have occurred only once. Mutations found in all samples of twin B’s tumour (cluster 4, **Figure 3C**) were clonal in her skin infiltrate but subclonal in her brain lesions (**Supplementary Figure 8**).

Tumour of twin B shows evidence of mutations which we have placed in clusters 1, 2, 3, 4, 5 and 9. Because clusters 4-5 and 9 are parallel and cannot be nested within each other, this indicates that the metastasis was not monoclonal (i.e., it was contributed by cells from at least two different lineages).

We considered whether there were one or more metastatic events from twin A to twin B. While we have not sampled the whole tumour of twin B, the data from samples available to us is consistent with a single metastatic event. In both the brain and the skin, we find evidence of the same lineages (marked by mutation clusters 1-5 and 9), with lower contributions of cluster 9 than 1-5 in both the brain and the skin. Achieving the same clonal composition in two independent metastases is unlikely, although it cannot be excluded.

Our data are consistent with tumour spread within twin B occurring from the brain to the skin. In both brain samples, the VAFs of mutations in cluster 4 (twin B-specific) are significantly lower than mutations in the cluster of truncal mutations (**Figure 3C**), while in the skin, the VAFs of mutations in those clusters do not significantly differ. If the metastasis occurred from the skin to the brain, the VAFs of those mutations would have been expected to be clonal also in the brain. However, it is important to note that we cannot exclude metastases from other regions of the skin, and only sampling the whole of the tumour would unambiguously resolve this question.

**4 Timing of tumour origin in twin A and spread to twin B**

We timed the transfer of the tumour from twin A to twin B by quantifying the mutations along the tumour phylogeny. The longest tumour branch was 120 substitutions, comprising 113 tumour variants and seven twin A-defining mutations (**Fig 3C-E, Supplementary Data S11**). As 91 variants are shared by both twins’ tumours, the tumour spread at ~75% (91/120) of its mutational age (although we cannot exclude earlier spread due to undetectable subclonal mutations). To estimate when the cancer’s cell of origin ceased to be a normal cell, we timed the generation of the *MN1::ZNF341* driver fusion. As the fusion is clonal it must have occurred before the end of the tumour trunk, which is composed of 31 clonal substitutions and seven embryonic mutations. It occurred, therefore, during the first third of the tumour’s mutational lifespan ( (31+7)/120). Those estimates provide a relative timing of tumour origin and spread, although we note that we are unable to detect subclonal mutations present at a very low VAF, which may impact the accuracy of our estimates.

Mutational times may be converted into real times if we assume a linear mutation rate. This assumption is supported by two observations: first, the mutation burden of the tumour is low compared to other childhood cancers (**Supplementary Figure 9**); and second, the trinucleotide context of these substitutions is consistent with endogenous processes that are thought to act as cellular clocks (signatures 1, 5, and 40a)^,2^ (**Supplementary Figure 10**). Accordingly, the gene fusion arose in the first trimester (38/120 variants × 40 weeks (the age of twin 2 at the time of sampling) = ~13 weeks) whilst tumour spread occurred during the second (91/120 variants × 40 weeks = ~26 weeks).

**SUPPLEMENTARY FIGURES**

**Supplementary Figure 1**. Reconstruction of the fusion from bulk RNA-seq reads from the twin A liver sample (contaminated with tumour). **Panel A** shows the view of RNA-seq reads of ZNF341, including split reads that map to MN1 exon 1. **Panel B** shows the view of RNA-seq reads of ZNF341, including split reads that map to ZNF341 exon 2.

**Supplementary Figure 2**. Estimates for variant allele frequencies (VAF) of the early embryonic mutations in each normal sample**.** Error bars show 95% confidence intervals derived from the binomial distribution based on the sequencing depth. Mutations labels (A-T) follow the names in Figure 2 and Supplementary Data 3. Mutations are grouped and colored based on their presence in the normal tissues of both twins, with mutation A present in both twin A and twin B, mutations B-H found exclusively in tissues of twin A, and mutations I-T specific to twin B. Source data are provided as a Source Data file.

**Supplementary Figure 3.** Estimates for variant allele frequencies (VAF) of the early embryonic mutations in each placental sample (A - bulk placental samples, B – LCM placental samples enriched for trophoblast)**.** Error bars show 95% confidence intervals derived from the binomial distribution based on the sequencing depth. Mutation names and descriptions as in Supplementary Figure 2. Source data are provided as a Source Data file.

**Supplementary Figure 4.** Estimates of the contribution of each major lineage marked by a group of mutations to each twin and the placenta. Error bars show 95% confidence intervals derived from the binomial distribution based on the sequencing depth and number of mutant reads of all mutations matching a corresponding to all mutations that marked the specific lineage. Mutation names and descriptions as in Supplementary Figure 2. Source data are provided as a Source Data file.

**Supplementary Figure 5.** Estimates for cancer cell fractions (CCF) of the early embryonic mutations in the tumour in comparison to normal tissues**. Panels A**-**B** present VAFs of early embryonic mutations in twin (**A**) and twin B tumour (**B**) compared to variant allele frequencies across other tissues of each twin. Samples of spleen of twin A and skin of twin B are excluded from the aggregate values of normal samples due to contamination with mutations from the other twin. **Panel C** presents CCFs of early embryonic mutation in each tumour sample. Mutation names and descriptions as in Supplementary Figure 2. Error bars show 95% confidence intervals derived from the binomial distribution based on the sequencing depth with the conversion from VAF to CCF. Source data are provided as a Source Data file.

**Supplementary Figure 6**. Clustering of tumour specific mutations. Each vertical column represents one mutation. The colour scale represents CCF. Cluster labels 1-13 correspond to numbering on Figure 3C. Source data are provided as a Source Data file.

**Supplementary Figure 7**. Mean cancer cell fractions of each cluster of tumour specific mutation in each normal and tumour sample of both twins and the placenta (bulk). Source data are provided as a Source Data file.

**Supplementary Figure 8.** Comparison of mutations in cluster 4 (specific to twin B tumour) and cluster 1 (truncal) in twin A and twin B samples. Error bars show 95% confidence intervals derived from the binomial distribution based on the sequencing depth. Source data are provided as a Source Data file.

**Supplementary Figure 9. Comparison of tumour mutation burden.** The red line indicates the mutation burden observed in the tumour reported here. SNV – single nucleotide variant. Data for comparison are from Thatikonda et al.^3^

**Supplementary Figure 10**. Trinucleotide mutation spectrum of 212 tumour-specific mutations. **Panel B** shows the relative activity of signatures identified in this set of mutations (SBS1, SBS5, SBS40a). The spectrum of tumour mutations was decomposed to known signatures using SigProfiler Assignment with cosmic version 3.5 and excluding signature groups which were biologically implausible (e.g., signatures associated with drugs and environmental exposures).

**Supplementary Figure 11. Estimation of twin-twin transfusion. Panels A**-**B** show variant allele frequencies of early embryonic mutations in twin A spleen (**A**) and twin B spleen (**B**) compared to variant allele frequencies across other tissues of each twin. Mutation names and descriptions as in Supplementary Figure 2. Error bars show 95% confidence intervals derived from the binomial distribution based on the sequencing depth. Source data are provided as a Source Data file.

**Supplementary Figure 12. Models of monozygotic twinning.** **Panel A** compares two of the possible models of twinning. Left: classical model of monozygotic twinning proposed by Corner^5^. Monozygotic twins develop from the splitting of the blastocyst at days 5-6 post-conception. Right: model of monozygotic twin formation where the two twins form through the separation of cells at the first cleavage division^6^. The first model predicts that the early developmental lineages will be present in both twins, while the second model predicts no sharing. **Panel B** shows the possible impact of cell death on the estimation of clonal composition of both twins. One could propose a scenario where each twin is formed from two cells from each lineage (marked in red and blue), but due to cell death, the red cells constitute ⅔ surviving cells of twin A, while blue lineage contributes ⅔ of twin B, leading to apparent asymmetries. While we cannot exclude this scenario, the probability of cell death in the early embryo is thought to be low (< 5%) and only notable after the 64 cell-stage^7^, therefore it is unlikely to have had a major impact on our estimates.

**Supplementary Figure 13. Filtering steps to obtain a set of high-quality somatic mutations.** The top plot shows all mutations (n = 10,954) retained after initial filtering or germline mutations. From top to bottom, we show trinucleotide contexts of mutations retained after each subsequent filtering step. To the right, we show trinucleotide context spectra of mutations removed after each filtering step. Numbers in brackets indicate the number of mutations in a given set. X axis labels as per Extended Data Figure 10. Source data are provided as a Source Data file.

**Supplementary Figure 14 Filtering out germline mutations present on regions of altered copy number in the germline).** A. Clusters of mutations identified as germline from binomial test (‘germline’), retained mutations present in all normal samples and retained mutations not identified in all normal samples. Mutations present in all normal samples are present in a cluster that exhibits a higher coverage than other classes of mutations, suggesting they could be associated with copy number changes in the germline. B. Labelled mutations excluded following filtering out mutations with coverage in aggregated normal samples > 1.25x median or < 0.75x median of aggregated coverage in normal samples for all mutations identified on a given chromosome. C. Labelled mutations excluded following filtering out mutations with coverage in aggregated normal samples > 1.25x median or < 0.75x median of aggregated coverage in normal samples for all mutations identified on a given chromosome, or present in clusters of > 10 mutations in 50kb region. D. Validation of filtering. We plotted all 1,069 mutations retained in the analysis following application of the first filters and plotted their coverage in aggregated normal samples against the distance to the nearest mutation (log10). 542 mutations labelled as likely germline show lower distance to the nearest mutation and differences in coverage. Source data are provided as a Source Data file.

**Supplementary Figure 15. Estimates of tumour cell fraction in normal and tumour samples based on evidence of copy number alterations. Panel A** displays estimates of purity in each normal sample. **Panels B and C** present VAF of chromosome 1p and 18q in each normal sample from twin A (B) or twin B (C). Only the portion of the chromosome 1 or 18 which was lost in the posterior fossa tumour sample from twin A is shown, as we were only able to phase heterozygous SNPs located on this chromosome segment. Each point shows the median VAF of mutations present on either chromosome in a bin of 1 Mb. Source data are provided as a Source Data file.

**Supplementary Figure 16. Estimates of tumour cell fraction in normal and tumour samples based on VAFs of early embryonic vs tumour-specific mutations. Panel A** Panel B shows estimates of the content of normal and tumour cells in each sample, based on the median VAF of 26 mutations classified as tumour-specific in this sample. C-D. VAF of 34 mutations in normal samples from twin A (C) and twin B (D). Mutations labelled as tumour specific or shared embryonic based on VAF in normal samples. In normal samples not infiltrated by tumour cells, VAF of tumour-specific mutations is 0. Source data are provided as a Source Data file.

**SUPPLEMENTARY REFERENCES**

1. Tan SY, Evans MG, Saab R, et al. Aggressive Pediatric Primitive Round Cell Tumors with MN1::ZNF341 Fusion: A Mimic of Neuroblastoma. *Pediatr Blood Cancer*. 2025;72(1):e31425. doi:10.1002/pbc.31425
2. Wang, Y. *et al.* Systemic mutagen exposures reported by normal kidney cell genomes. 2026.04.07.716715 Preprint at https://doi.org/10.64898/2026.04.07.716715 (2026).
3. Thatikonda V, Islam SMA, Autry RJ, et al. Comprehensive analysis of mutational signatures reveals distinct patterns and molecular processes across 27 pediatric cancers. *Nat Cancer*. 2023;4(2):276-289. doi:10.1038/s43018-022-00509-4
4. Díaz-Gay, M. *et al.* Assigning mutational signatures to individual samples and individual somatic mutations with SigProfilerAssignment. *Bioinformatics* **39**, btad756 (2023).
5. Corner, G. W. The observed embryology of human single-ovum twins and other multiple births. *Am. J. Obstet. Gynecol.* **70**, 933–951 (1955).
6. Herranz, G. The timing of monozygotic twinning: a criticism of the common model. *Zygote* **23**, 27–40 (2015).
7. Junyent, S. *et al.* The first two blastomeres contribute unequally to the human embryo. *Cell* **187**, 2838-2854.e17 (2024)
